# Supplementary material for: All-at-once RNA folding with 3D motif prediction framed by evolutionary information
Source: Nat Methods. 2025 Oct 3;22(10):2094–106. doi: 10.1038/s41592-025-02833-w (PMC12510878; doi:10.1038/s41592-025-02833-w)
Supplement: Supplementary file 3 — Source code to run CaCoFold-R3D. Data and results. [file 41592_2025_2833_MOESM3_ESM.gz › supplemental_material/results/CaCoFold-R3D/CaCoFold-R3D_Rfam_vshuffle/RF01136_sR28.R2R.sto.pdf]

RF01136_sR28

5′ – R ● ● ● A U G A U G A ● Y G G Y U U C G G G ● C Y G A R ● G R U G A ● G A C A Y C A G C U A U C G C U G A ● ●
